# Immune checkpoint inhibitors in ovarian cancer: where do we go from here?

**DOI:** 10.20517/cdr.2023.13

**Published:** 2023-06-14

**Authors:** Won-Hee Yoon, Anna DeFazio, Lawrence Kasherman

**Affiliations:** ^1^Department of Medical Oncology, Blacktown Cancer and Haematology Centre, Blacktown Hospital, Blacktown 2148, Australia.; ^2^Department of Medical Oncology, Crown Princess Mary Cancer Centre, Westmead Hospital, Westmead 2145, Australia.; ^3^Centre for Cancer Research, The Westmead Institute for Medical Research, Westmead 2145, Australia.; ^4^Faculty of Medicine and Health, The University of Sydney, Camperdown 2050, Australia.; ^5^Department of Gynecological Oncology, Westmead Hospital, Westmead 2145, Australia.; ^6^The Daffodil Centre, The University of Sydney, a joint venture with Cancer Council New South Wales, Sydney 2011, Australia.; ^7^Department of Medical Oncology, Illawarra Cancer Care Centre, Wollongong 2500, Australia.

**Keywords:** Ovarian cancer, immunotherapy, tumour microenvironment, drug development

## Abstract

Epithelial ovarian cancer (EOC) is the most lethal gynaecological malignancy, and despite advancements in therapeutics, most women unfortunately still succumb to their disease. Immunotherapies, in particular immune checkpoint inhibitors (ICI), have been therapeutically transformative in many tumour types, including gynaecological malignancies such as cervical and endometrial cancer. Unfortunately, these therapeutic successes have not been mirrored in ovarian cancer clinical studies. This review provides an overview of the ovarian tumour microenvironment (TME), particularly factors associated with survival, and explores current research into immunotherapeutic strategies in EOC, with an exploratory focus on novel therapeutics in navigating drug resistance.

## INTRODUCTION

Ovarian cancer is one of the most lethal cancers worldwide, with over 200,000 deaths in 2020^[1]^_._ Epithelial ovarian cancer (EOC) is the most common subtype (90%), with germ cell tumours, sex cord-stromal tumours and other rare subtypes, including small cell carcinoma, accounting for the remaining 10%^[2]^. EOC (including fallopian tube and primary peritoneal cancer) is a complex disease as it encompasses a heterogenous group of inherently different histological subtypes with differing underlying genomic and molecular drivers resulting in different clinical behaviours and outcomes^[3,4]^_._ High-grade serous ovarian carcinoma (HGSOC) is the most common, followed by endometrioid carcinoma, clear cell carcinoma, low-grade serous carcinoma, and mucinous carcinoma^[2]^.

Most women with EOC are diagnosed at advanced stage and more than 70% will recur within the first three years. *BRCA1/2* germline mutations are the strongest known genetic risk factors, with an estimated frequency of 13%-16% in women diagnosed with EOC^[5,6,7]^, most of which are HGSOC histological subtype^[7]^. Germline *BRCA1/2* mutations are associated with greater sensitivity to platinum-based chemotherapy and poly(adenosine diphosphate[ADP]-ribose) polymerase inhibitors (PARPi), and carriers have generally been reported to have improved survival compared to non-mutation carriers with high-grade serous EOC^[8,9]^_._

The majority of EOC patients will eventually succumb to the disease, with 10-year survival being only 17%^[10,11]^. Cytoreductive surgery with chemotherapy remains the standard of care in the treatment of EOC^[12]^. Doublet-platinum chemotherapy can be administered as adjuvant treatment or as a neoadjuvant regime (usually three cycles prior to interval debulking surgery, followed by the remaining three cycles). Neoadjuvant chemotherapy provides an opportunity to assess chemosensitivity and assists with prognostication^[13,14]^. Other drug classes such as vascular endothelial growth factor (VEGF) inhibitors have shown survival benefits, and thus bevacizumab is routinely used in combination with chemotherapy as a maintenance strategy^[15-17]^. Most recently international, phase 3 studies have proven the effectiveness of PARPi in improving both progression and overall survival as well as the quality of life outcomes in both front-line and recurrent maintenance settings for certain subsets of advanced EOC, particularly those with *BRCA* mutations^[8,9,18,19]^_._ However, not all benefit from PARPi use, particularly those who are platinum-resistant, those without *BRCA* gene mutations and/or with intact homologous recombination repair (HRR) pathway, which comprises approximately 50% of the most common EOC histological subtype, HGSOC^[3]^. Unfortunately, even with PARPi, many patients will develop resistance and thus chemotherapy remains important in the treatment of recurrent ovarian cancer. With successive lines of treatment, most EOC will become resistant to chemotherapy, in particular to platinum-containing agents. Other subtypes such as low-grade serous, mucinous carcinoma and clear cell carcinoma are largely resistant to chemotherapy as a result of molecular and genetic profile and alterations^[20]^. Studies in preclinical models have suggested a number of mechanisms underlying acquired platinum resistance in EOC, including drug accumulation, drug efflux, cellular response to DNA damage and impaired apoptosis; however, evidence for clinical relevance for most of these mechanisms is lacking^[21]^. Limitations include using cell line models that are not representative of the common EOC subtypes seen clinically^[22]^, and using non-physiological drug exposures to induce drug resistance phenotypes *in vitro*. More compelling studies investigating patient samples following the development of clinical resistance *in vivo* have implicated restoration of DNA repair pathways, including reversion of *BRCA* mutations and methylation, as significant mechanisms of acquired platinum resistance^[23-25]^. As the outlook for platinum-resistant EOC remains extremely guarded, with a median survival of only 12 months^[26]^, there has been a strong focus on identifying mechanisms of resistance against platinum chemotherapy and PARPi, to address this urgent unmet therapeutic need. As such, many clinical trials are now standardly incorporating exploratory correlative studies with emerging and innovative technologies to further clarify molecular signalling events and proteomic characterisation of ovarian cancer to gain a deeper understanding of the disease, with the ultimate goal of developing and incorporating novel therapeutics to avoid and/or bypass mechanisms of intrinsic and acquired resistance in EOC.

Immunotherapy has been transformative in the oncology therapeutic landscape. Immune checkpoint inhibitors (ICIs) counter several major immune-evasion mechanisms of cancer to induce killing of tumour cells by CD8+ T cells. Dramatic effects of improved progression-free and overall survival with utilisation of ICI have been demonstrated in a number of cancers, including melanoma, lung and colorectal cancers^[27-29]^. The durability and sustained benefit of ICI in other tumours make it an attractive therapy in ovarian cancer, particularly when there is evidence that ovarian cancer is immunogenic with the presence of tumour-infiltrating lymphocytes (TILs), regulatory T cells (Tregs), natural killer cells and tumour-associated macrophages (TAMs) detected in peripheral blood, ovarian cancer tissue and ascites^[30-33]^. Specific immune cell signatures, combined with multiple co-occurring DNA repair gene alterations and increased predicted neo-antigen load, have been associated with response to treatment and exceptionally long survival in patients with HGSOC, providing further evidence for tumour immune activity, at least in a proportion of cases^[34]^_._

However, disappointingly, durable responses to single-agent ICI in platinum-resistant EOC remain modest^[35-37]^ and published studies to date have returned negative results with the addition of programmed cell death receptor-1 (PD1/L1) targeted ICI to standard adjuvant chemotherapy with or without bevacizumab^[38,39]^. Intriguingly, more encouraging clinical trial results from early phase studies exploring combination ICI with other agents such as PARPi, and VEGF inhibitors to act as immunomodulators have emerged.

In this article, the authors will describe the interplay between the immune system and tumour microenvironment (TME) including its interaction with the DNA damage repair (DDR) pathway, summarise the clinical trial landscape of immunotherapy in EOC, and discuss novel therapeutic pathways under investigation to overcome ICI therapy resistance.

## ROLE OF THE TUMOUR MICROENVIRONMENT IN EPITHELIAL OVARIAN CANCER

As previously mentioned, ovarian cancer encapsulates a heterogenous group with subtypes that differ histologically, genomically and molecularly^[3]^. A dualistic model categorising EOC into two groups, Type I and Type II EOC, has been described^[3]^. According to this classification, Type I includes low-grade subtypes, typically arising from a recognisable precursor lesion such as borderline tumours with low malignant potential, whereas Type II EOC includes HGSOC, the most common subtype, with frequent *TP53* mutations and HRR pathway gene alterations^[40]^. Immune-cell infiltrates differ markedly between the histological subtypes, with the high-grade serous showing the most TIL infiltration, and the other subtypes being largely “cold” with respect to immune cell infiltrates, although there is wide variability between individual patients^[41,42]^.

The defining genomic features of HGSOC are profound structural variation, including gene copy number alterations and genomic rearrangements, on a background of near-ubiquitous *TP53* mutation, with the most common somatic and germline alterations found in HRR pathways genes, mainly *BRCA1* and *BRCA2*^[23]^. When lacking HRR function, as in BRCA-mutant cells, DNA double-strand breaks will be processed by alternative but error-prone repair pathways, such as the non-homologous end joining repair (NHEJ), which lead to the accumulation of genomic instability and ultimately cancer cell death. NHEJ is faster than homologous recombination and mainly occurs in the G1 phase. Nevertheless, there is recent evidence that NHEJ functions throughout the cell cycle. Beyond the already-known proteins, such as Ku70/80, DNA-PKcs, Artemis, DNA pol λ/μ, DNA ligase IV-XRCC4, and XLF, new proteins are involved in the NHEJ, namely PAXX, MRI/CYREN, TARDBP of TDP-43, IFFO1, ERCC6L2, and RNase H2. Among them, MRI/CYREN has a dual role, as it stimulates NHEJ in the G1 phase of the cell cycle and inhibits the pathway in the S and G2 phases^[43]^. In addition to this genomic complexity, a challenging clinical feature of HGSOC is the presence of widespread intraperitoneal disease at the time of diagnosis. Distinct spatial immunostimulatory and immunosuppressive mechanisms have been identified within individual patients depending on the anatomical site, with a relative paucity of immune cells in primary adnexal disease sites, in contrast with metastatic sites^[44]^.

The impact of the heterogeneity of the tumour microenvironment on response to immunotherapy between and within ovarian cancer patients is not yet fully understood.

**Figure 1:**
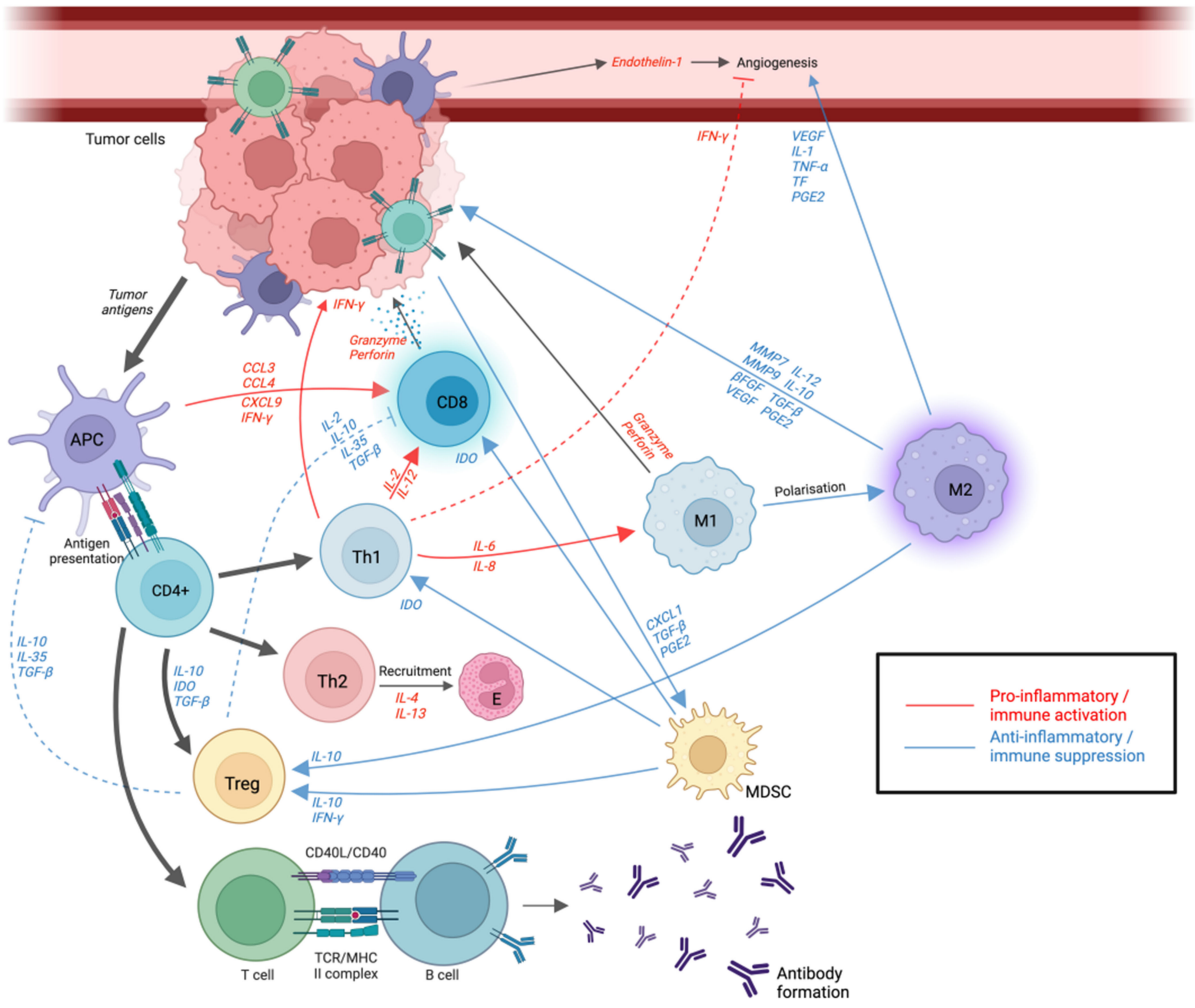
Schematic diagram outlining complex pro- and anti-inflammatory interplay within epithelial ovarian cancer microenvironment. Created with Biorender.com. Tumour cells recognised by APC, from which tumour antigens are presented to naïve CD4+ T cells, triggering T cell differentiation into Th1, Th2, Treg cell subclones (and other Th subsets – not pictured). Cytotoxic CD8+ T cells are recruited to TME via chemokines such as CCL3 and CXCL9 and are activated. Once activated, it releases granzyme and perforin to lyse cells directly. Th1 cells have dual action by secreting cytokines including IL-2 and IL-12, further activating CD8+ T cells to directly kill tumour cells as well as secreting IFN-gamma to suppress angiogenesis. Th2 assists eosinophil recruitment via IL-4 and IL-13 cytokine release, which assist in antitumour response. Treg cells suppress DC maturation and mute CD8+ T cell response by secreting inhibitory cytokines IL-10, IL-35, and TGF-β in response to regulate inflammatory response. M1 TAM, a pro-inflammatory type, is involved in tumour destruction, while M2 TAM exhibits immunosuppressive effects allowing tumour migration and invasion by releasing factors including endothelin-1, VEGF and TNF-alpha to induce tumour angiogenesis. CD4^+^ T cells bind to B cells via major histocompatibility II complex to induce antibody formation via the adaptive immune system. APC: antigen-presenting cell; MDSC: Myeloid-derived suppressor cells; IL: Interleukin; TGF-β: transforming growth factor-beta; TNF: Tumour necrosis factor; CCL: Chemokine (C-C Motif); CXCL: Chemokine (C-X-C Motif); IDO: Indoleamine 2,3-dioxygenase; Th: T-Helper; M1 and M2 TAM: Tumour-associated macrophages; MMP: matrix metalloproteinase; E: eosinophil; VEGF: vascular endothelial growth factor; TF: tissue factor; PGE2: prostaglandin E2; IFN: interferon; β-FGF: beta-fibroblast growth factor.

The immune system plays an integral and extensive role in tumour formation and growth in all tumours, including ovarian cancer [Figure 1]. Tumour growth is facilitated by the critical interplay between TME and immune cells, including natural killer (NK) cells, CD8+ T cells and CD4+ helper T (Th) cells together with pro-inflammatory macrophages (M1) and dendritic cells. When a tumour is formed, it is recognised and eliminated by the immune system (elimination phase). The tumour cells which survive this process enter a phase called equilibrium phase, during which the tumour can continue to grow despite ongoing destruction from the immune system. Eventually, tumour cells evade the immune recognition and destruction, termed the escape phase, leading to continual tumour growth and progression and finally metastasis. TME is a collective term that encapsulates a dynamic interplay between tumour cells and components of the immune system and plays an important role in tumour differentiation, dissemination, and immune evasion. It consists of extracellular matrix consisting of matrix metalloproteinases (MMPs) and stromal cells including tumour-infiltrating lymphocytes (TILs), fibroblasts, and endothelial cells, amongst others.

### Tumour-infiltrating lymphocytes

TILs are white blood cells including T cells, B cells, macrophages and natural killer cells, which are localised to tumour or tumour stroma in response to molecular signals. CD3+, CD4+ ,and CD8+ TILs have long been known to exist in ovarian cancer, and an association between the presence of TILs and improved overall survival (OS) in ovarian cancer has been demonstrated in multiple studies. A meta-analysis to evaluate the prognostic value of TILs in ovarian cancer has found a significant association between TILs and survival, with a hazard ratio of 2.24 for those without TILs^[45]^_._ A study of 5577 EOC tissue samples identified that CD8+ TILs were associated with longer OS for those with multiple histological subtypes, particularly those with high-grade serous ovarian carcinomas. The median OS survival was 2.8 years for those with no CD8+ TILs compared to 5.1 years in those with high levels regardless of the extent of residual disease post cytoreductive surgery, standard treatment, and germline *BRCA1* mutation^[41]^. It also demonstrated prognostic value in other histological subtypes including endometrioid and mucinous carcinomas. Overall, CD8+ TILs have become the standard for prognostic evaluation for TILs in ovarian cancer.

### CD8+ T lymphocytes

When naïve T cells are activated, they differentiate into CD8+ cytotoxic T cells and are migrated into TME via a number of signalling proteins called chemokines (including CCL5, CXCL9, CXCL10)^[46]^. CD8+ cytotoxic T cells target tumour cells via T cell receptor interaction with MHC Class I, facilitating tumour apoptosis via several mechanisms including perforin and granzyme B secretion. Tumour cells adopted numerous mechanisms to evade apoptosis, such as downregulation of MHC Class I, dysregulated expression of death receptors, or modification of antigen processing and thus reduced presentation capacity^[47]^. Additionally, CD8+ cytotoxic T cells upregulate checkpoint receptors such as PD-1, CTLA-4, LAG-3 and TIM3, which makes them susceptible to inhibitory signalling since binding of checkpoint receptors induces cytotoxic T cell exhaustion and anergy^[47,48]^.

### CD4+ T lymphocytes

Naïve CD4+ T cells are activated by tumour antigens, and they differentiate into different subsets, including Th1, Th2, Th9, Th17, Th22, and Tregs. While the majority of CD4+ lymphocyte subsets further assist the activation of CD8+ T lymphocytes, thus facilitating the killing of the tumour, some have dual roles in promoting tumour growth via increasing angiogenesis.

### T regulatory cells

T regulatory cells (Tregs) play an important role in suppressing immune responses and maintaining self-tolerance. Tregs in ovarian cancer are identified by several marker expressions, CD4 and CD35, which are present extracellularly, or forkhead box P3 (FOXP3) located intracellularly. When activated, Tregs release inhibitor cytokines such as TFT-β and IL-10 facilitating immunosuppressive effects on TME^[49]^. Increased Treg presence has been identified in tumours that are able to evade immune destruction, and it has been demonstrated that increased recruitment of Treg cells is associated with reduced survival with a high death hazard ratio in ovarian cancer^[45,48]^. Those who have undergone primary debulking surgery for ovarian cancer were identified to have decreased Tregs and increased TILS compared to those with suboptimal debulking surgery had the opposite trend^[50,51]^_._ Additionally, those undergoing neoadjuvant chemotherapy had lower FOXP3+ Treg infiltration and demonstrated higher survival compared to those with higher FOXP3 Treg counts^[52]^_._ Although Treg is not recognised as a prognostic factor, it seems clear that Tregs in TME have immunosuppressive effects, thus hampering the immune system to destroy cancer cells.

### Myeloid-derived suppressor cells

Myeloid-Derived suppressor cells (MDSCs) consist of a heterogenous population of myeloid cells expressing GR-1 and CD11b myeloid surface markers. MDSCs mostly act to suppress the immune system via T cells in multiple ways, eventually promoting tumour progression. MDSCs reduce essential amino acids such as L-arginine and L-cystine required for T cell activation and function^[53]^, and also inhibit the recruitment of T cells and promote T cell apoptosis. MDSCs also promote Treg cell activation and stimulate oxidative stress as well as facilitate neovascularisation, therefore priming and promoting tumour progression^[53,54]^.

### Dendritic cells

Dendritic cells (DCs) play a critical role in bridging the innate and adaptive immune systems and are essential in precipitating T cell immune response. As antigen-presenting cells, DCs facilitate tumour antigen recognition and presentation, thus triggering appropriate T-cell response. DCs also play an important role in activating and manipulating cytotoxic T-lymphocyte population in the TME mostly mediated by appropriate DC maturation. When DC maturation process becomes faulty due to various mechanisms, it can result in a more tumour-tolerant TME, thereby promoting tumour progression.

### Programmed cell death ligand-1

Up to 60% of EOC express PD-L1, which has been identified as a poor prognostic factor for both PFS and OS^[37-39,55,56]^. In a study by Hamanishi *et al.*, five-year survival rates were found to be 80.2% *vs.* 52.6% for high and low PD-L1 expressing EOC, respectively^[56]^. Furthermore, CD8+ TILs appear to be inversely correlated to PD-L1 expression^[56]^. PD-1 is a surface molecule commonly expressed on CD8+ T cells and is a negative regulator of T cell activation. Its expression blocks entry into the cell cycle and production of cytokines such as IFN-gamma and TNF-a involved in activating inflammatory and immune responses^[57]^. Upregulation of PD-L1, which is exclusively expressed on the surface of tumour cells and a common ligand for PD-1, causes impaired tumour destruction and apoptosis of T cells. PD-1 pathway activation also escalates Treg function^[58]^. Thus, collectively activation of the PD-1/L1 pathway collectively results in the ability of cancer cells to evade the immune system and facilitating ongoing tumour growth. Harnessing and manipulating this pathway has been the rationale for therapeutic use for PD-1/L1 targeted ICIs. Despite the tremendous efficacy and clinical benefit of anti-PD-1/L1 ICI in other tumour types, it has been largely disappointing in ovarian cancer.

## IMMUNE CHECKPOINT INHIBITORS: A WORK IN PROGRESS

Current understanding of immune-mediated response in relation to malignancy and tumour microenvironment has exponentially increased over the last two decades, especially with the development of immune checkpoint inhibitors. ICIs are now being commonly utilised in the treatment of both non-solid and solid malignancies, with efficacy rates ranging from 70% in lymphoma^[59]^ and 39%-60% well-selected solid tumour groups, including those with high microsatellite instability (MSI-H)^[60,61]^. Impressively, in some cohorts, such as metastatic melanoma, 22% and 19% were able to achieve complete response with doublet and single-agent ICI, respectively^[27]^. Additionally, the durability of ICI makes it an attractive therapeutic, with overall survival of 44%-52% in metastatic melanoma cohorts at five-year follow-up noted^[27]^. Unfortunately, these drugs are not without their side effects. While reasonably well tolerated, grade ≥ 3 treatment-related adverse events occur in approximately 20%-25% of cases, and this rate can increase to 59% with the use of doublet ICIs^[27,28]^. While most immune-related toxicities are easily manageable, they are often life-long issues that require constant monitoring and management; thus, careful selection of patients for immunotherapy is paramount^[27]^. Additionally, the cost of ICI is problematic, as most Western and first world countries can fund it via government-subsidised programs or medical insurance; generalisability and utility of ICI worldwide realistically will be challenging. Particularly in low-middle-income countries, financial toxicity is a real issue affecting the quality of life for many patients affected by cancer.

**Figure 2:**
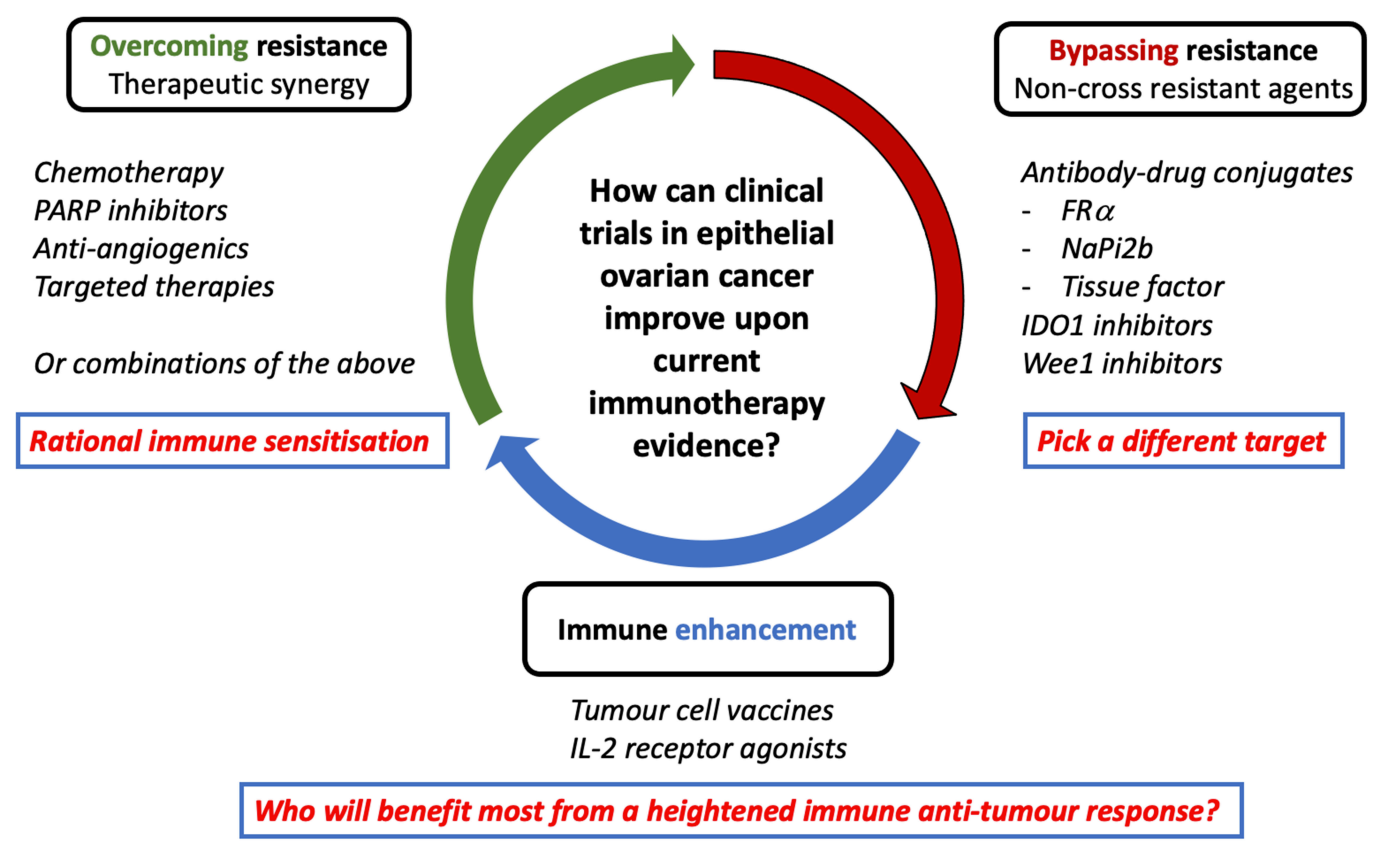
Schema outlining biomarker-driven strategies to improve upon current evidence for immunotherapy in ovarian cancer. Current trials are focused on improving clinical outcomes by either overcoming resistance through synergistic therapeutics (rational immune sensitisation) or bypassing resistance mechanisms (choosing different therapeutic targets). FRα: folate receptor-alpha; IDO1: Indoleamine 2, 3-dioxygenase 1; PARP: Poly-ADP ribose polymerase.

Despite the significant advances in the understanding of the role of TME in EOC, the efficacy of single-agent and combination ICI therapy has been somewhat disappointing, as demonstrated through numerous international randomised trials. Amongst tumour types where immunotherapy is typically effective, there have been therapeutic strategies posited to transform “cold” tumours to “hot”, that is, by increasing T-cell infiltration to predispose tumour cells to immune therapy anti-cancer effects^[62]^. Given the current therapeutic landscape of ovarian cancer and the limited role of immunotherapy, ongoing clinical trials are focused generally on three mechanisms: overcoming resistance by administering ICI therapy in synergism with other drug classes, enhancing immune responses through utilising other immune pathways, or bypassing resistance through exploring alternative therapeutic pathways [Figure 2]. Biomarker studies will provide further crucial information in determining which patients will benefit from particular treatment strategies. This section outlines the state of evidence for ICI therapies in EOC and discusses combination trials in progress looking to overcome resistance.

### Single-agent ICI therapy

Several PD-1/L1 targeted ICI monotherapy trials exist in advanced or recurrent EOC, most of which demonstrated modest efficacy. KEYNOTE-100 study, a phase II trial which included a total of 376 advanced recurrent EOC patients administered 200 mg of pembrolizumab, 3-weekly^[63]^. It demonstrated only a modest ORR of 9.9% with a suggestion of the trend of increased responses for those with higher PD-L1 expression measured by combined positive score (CPS). The median PFS was 2.1 months, while the median OS was 18.7 months. Aside from CPS, no other clinical features such as histology or platinum-sensitivity display a correlation with higher response.

Numerous other studies investigating different single-agent anti-PD-1/L1 ICI agents such as avelumab and nivolumab in recurrent and platinum-resistant disease have demonstrated similarly disappointing modest responses with ORR^[64-66]^_._ Despite the initial suggestion of higher response for clear cell carcinomas, this has been largely disappointing, with no clinically meaningful ORR or survival benefit in the recently presented MOCCA trial^[67]^. Additionally, JAVELIN Ovarian 100^[38]^ and IMagyn050^[39]^ incorporating ICI with standard chemotherapy in the upfront setting have demonstrated no significant benefits, without improvement in PFS or OS, despite PD-L1 stratification^[38,39]^_._

Despite disheartening single-agent anti-PD-1/L1 ICI results across the board of different EOC subtypes, a number of different studies examining different therapeutic combinations with ICI have been undertaken to investigate whether drug synergism of ICI can be enhanced for advanced EOC.

### Dual-agent ICI therapy

Ipilimumab is a monoclonal antibody targeting cytotoxic T-lymphocyte associated protein-4 (CTLA-4), another immune checkpoint surface protein involved in negatively regulating T-cell function in priming immune response. Combining anti-CTLA4/anti-PD1 inhibitors have demonstrated significant treatment effects in other solid cancers, although this efficacy is limited in EOC. Zamarin *et al.* demonstrated that from the 100 recurrent EOC patients, those who had doublet-ICI demonstrated higher ORR at 6 months (31.4% *vs.* 12.2%) and PFS improvement (HR 0.528, 95%CI: 0.339-0.821; *P* = 0.04)^[68]^. Again, there was no association between the magnitude of PD-L1 staining and other clinical outcomes, which suggests the need for better predictive biomarkers to assist with ICI usage guidance. As expected, grade 3 adverse events were higher in the combination group (49% *vs.* 33%), although this did not reach statistical significance.

### Combination studies with antiangiogenic agents

Angiogenesis is a hallmark of cancer growth and metastasis, and VEGF plays an integral role in this process. It binds to a number of VEGF receptor tyrosine kinases, namely VEGF-A, VEGF-B, VEGFR-C/D, VEGFR-1/2, and VEGFR-2/3. Amongst solid cancers, EOC is known to have high VEGF expression^[69]^ and the use of bevacizumab, a VEGF-A inhibitor, is commonplace with proven clinical benefit in both platinum-sensitive and platinum-resistant settings^[17,70]^. Furthermore, there is strong evidence demonstrating the immunomodulatory effects of VEGF; preclinical data demonstrate that signalling through VEGFR-1 mediated by VEGFR-A can suppress maturation of dendritic cells, increase Treg population via VEGFR-2 signal and stimulate the growth of myeloid-derived suppressor cells, leading to further suppression of TME^[71,72]^. VEGF blockade leads to increased cytotoxic T cell recruitment and migration to tumour cells with a reduction in CD4+ Treg in TME^[50,73,74]^_._ Therefore, the potential synergistic activity of ICI therapy with antiangiogenic therapy has been hypothesised.

In addition to the previously discussed IMagyn050 in the upfront setting, nivolumab and atezolizumab combined with bevacizumab have been investigated in the recurrent setting^[68,75]^_._ Liu *et al.* recruited 38 women with recurrent, pre-treated EOC in a phase II study in which the ORR was higher than single-agent ICI with 28.9%; however, disappointingly, the majority of the benefit appeared to lie within the platinum-sensitive cohort with a median PFS of 12.1 months versus 7.7 months in the platinum-resistant cohort^[76]^. These findings again highlight the importance of patient selection for these therapies, although at present, aside from platinum sensitivity, more accurate and pertinent biomarkers are yet to be discovered to guide therapy.

Other antiangiogenic therapies have also been explored in combination with ICI therapy including lenvatinib and sitravatinib. Lenvatinib is a multi-receptor tyrosine kinase inhibitor, which mainly targets VEGF 1-3, fibroblast growth factor (FGF) receptors 1-4, platelet-derived growth factor receptor a, KIT, and RET. LEAP-005^[77]^, an ongoing multi-cohort phase II study, includes 31 recurrent EOC patients with three or more prior lines of treatment including bevacizumab (ClinicalTrials.gov, NCT02501096). The results were more encouraging - ORR was 32% with a median PFS of 4.4 months, and nearly a quarter of responders were platinum resistant. Sitravatinib, an orally available multi-tyrosine receptor inhibitor that acts on VEGF2, KIT and TAM receptors, has also been identified to modulate immunosuppressive influences on TME. An early-phase study investigating tislelizumab, an anti-PD-1 monoclonal antibody, with sitravatinib in 60 platinum-resistant EOC demonstrated an ORR of 26% with a median PFS of 4.1 months and a median OS of 12.8 months^[78]^. Unfortunately, these novel combination therapies report significant treatment-related adverse events requiring dose reductions or interruptions in significant proportions of patients. Although these combinations are promising in overcoming therapeutic resistance in recurrent EOC, issues of tolerance and quality of life remain important factors. Lastly, AK112, a novel anti-PD-1/VEGF-A bi-specific antibody, has been investigated in an ongoing Phase Ia/Ib study in platinum-resistant/refractory EOC^[79]^_._ The ORR was higher at 29.4%, with two spectacular responders noted: one with clear cell histology with prior ICI exposure and one with HGSOC. The toxicity profile was similar to sitravatinib, but tolerability was much better with manageable adverse events in comparison.

In summary, trials so far investigating ICI and angiogenic combination approach have demonstrated preliminary efficacy, which appears to be clinically meaningful, but balancing this with the challenge of managing adverse events represents the true dilemma in moving forward, particularly in terms of patient selection for therapy, appropriateness of dose reductions, and ongoing biomarker discovery. As the role of combination angiogenesis and immunotherapy agents grows in EOC treatment, clear guidelines on practical aspects and real-world data will become increasingly relevant in informing clinicians of best practices.

### Combination studies with PARP inhibitors

PARPi, namely olaparib, niraparib and rucaparib, have demonstrated breakthrough improvements in survival outcomes for individuals with EOC. Defects in the HRR pathway are strongly associated with the development of high-grade serous and endometrioid ovarian cancers, and it is in these patients with demonstrable HRR deficiencies or mutations that PARPi confer the greatest clinical benefit. Unfortunately, a substantial proportion of individuals develop resistance to PARPi, and it is in this population that therapeutic direction remains unclear. Named resistance mechanisms to PARPi in the literature include drug efflux transporters, decreased PARP trapping, replication fork stabilisation and restoration of HR repair in multiple ways including alteration of PARP1, reversion mutation in HRR genes, and loss of *BRCA1* promotor methylation^[80]^_._ Of these, HR reversion mutations and drug efflux transporters (such as *ABCB1* mutations) have been implicated most prominently in the literature^[81-87]^.

PARPi are inherently immunogenic by the mechanism of action, leading to a consequent increase in genomic instability and formation of neoantigens leading to immune detection via T cells^[88]^. Additionally, there is evidence to suggest that *BRCA1* deficiency may induce STING-dependent immune response by inducing type I interferon and pro-inflammatory cytokine production, providing some interconnected relationship between the therapeutic effect of PARPi and the immune response^[89]^. It has also been demonstrated that PARP inhibition inactivates GSK3 and upregulates PD-L1 in a dose-dependent manner in various cell lines, such as breast and pancreatic cells^[90,91]^. Consequently, T-cell activation is suppressed, resulting in enhanced cancer cell apoptosis^[90]^. While the complete immunomodulatory effect of PARPi on TME is not yet fully explained, several studies have shown promising synergistic effects. TOPACIO, a phase I/II study, examined niraparib with pembrolizumab in 62 patients with recurrent platinum-resistant or -refractory EOC^[92]^. The ORR was 18%, with the most common treatment-related adverse events being grade 3 anaemia and thrombocytopaenia. No correlation in efficacy with *BRCA* and HRD status, PD-L1 expression and prior bevacizumab was observed. While it did not meet its predefined endpoint, the combination showed meaningful activity compared with single agent-based therapy in the same population with resistant or refractory OC. Another study examined olaparib with durvalumab in 32 platinum-sensitive EOC patients with germline *BRCA1/2* mutations and 31 *BRCA* wild-type patients with the addition of bevacizumab^[93]^. The ORR was 71.9% for olaparib and durvalumab in *BRCA* mutated cohort, 34.4% in *BRCA* wild-type patients, and 87.1% in the triplet arm *vs.* 34.4% with doublet therapy. Mean PFS was also longer with triplet therapy (14.7 *vs.* 5.5 months). Overall, both triple and doublet therapy was well tolerated, with adverse events reported consistent with those previously reported for single drugs, and discontinuation of one or more drugs as a result of adverse events was greater with triplet therapy (16% *vs.* 6%).

Currently, there are conflicting results seen in trials investigating triplet therapy consisting of PARPi, antiangiogenics, and ICI. Zimmer *et al.* conducted a trial of cediranib, an oral anti-VEGFR1-3 agent combined with durvalumab and Olaparib, in 35 recurrent EOC patients with promising preliminary results of an ORR of 44%^[94]^. A phase II is currently underway, with results expected in 2025. Meanwhile, other two-phase II studies, OPAL and GINECO BOLD, demonstrated a lower-than expected-ORR of 17.9% and no benefit in PFS and OS in either platinum-resistant or platinum-sensitive cohort^[95,96]^_._ Eventually, when results from larger combination studies (demonstrated in Table 1) become available, careful comparisons between triplet therapy studies will need to be considered, particularly in terms of patient selection, rates of toxicity and efficacy outcomes, especially as specific drug combinations can have varying synergistic effects. This is especially applicable to the trials in the first-line setting, where improvements in PFS and OS are expected to be highest in the chemotherapy-naïve setting.

**Table 1:** Summary of ongoing immune checkpoint inhibitor combination trials in epithelial ovarian cancer. Taken from clinicaltrials.gov. (Accessed on 9th of January, 2023)

**Trial Identifier**	**Trial name**	**Phase**	**Setting**	***n***	**Treatment Arm**	**Endpoints**	**Expected completion**
NCT04417192	Olaparib Monotherapy and Olaparib + Pembrolizumab combination Therapy in Ovarian Cancer (OLAPem)	II	UpfrontHRD positive only	30	Cohort 1: OlaparibCohort 2: Olaparib, Pembrolizumab	ORR*AECRSPFSOS	December 2023
NCT03740165	Study of chemotherapy with pembrolizumab (MK-3475) followed by maintenance with Olaparib (MK-7339) for the first-line treatment of women with BRCA non-mutated advanced epithelial ovarian cancer (EOC) (MK-7339-001/ KEYLYNK-001/ ENGOT-ov43/GOG-3036	III	Upfront	1367	Pembrolizumab, Olaparib + Chemotherapy (carboplatin/paclitaxel) + Bevacizumab	PFS*OSOS (PD-L1 CPS > 10)PFS2AEQoLTTDpCR	May 2025
NCT03737643	Durvalumab treatment in combination with chemotherapy with bevacizumab, followed by maintenance durvalumab, bevacizumab and olaparib treatment in advanced ovarian cancer patients (DUO-O)	III	Upfront	1374	Durvalumab, Olaparib + chemotherapy (carboplatin/paclitaxel) + bevacizumab	PFS*OSPFS2QoLpCRORRDOR	May 2028
NCT03522246	A study in ovarian cancer patients evaluating Rucaparib and Nivolmab as Maintenance treatment following response to front-line platinum-based chemotherapy (ATHENA)	III	Upfront	1000	Rucaparib, Nivolumab	PFS*OSORRDOR	December 2030
NCT03602859	A phase 3 comparison of Platinum-based therapy with TSR-042 and niraparib versus standard of care (SOC) platinum-based therapy as first-line treatment of stage III or IV non-mucinous Epithelial Ovarian Cancer (FIRST)	III	Upfront	1405	Dostarlimab, Niraparib +/- chemotherapy (carboplatin/paclitaxel)	PFS*OSAEQoL	June 2026
NCT04679064	Trial of Niraparib-TSR-042 (dostarlimab) *vs.* Physician’s choice of Chemotherapy in recurrent, ovarian, fallopian tube or peritoneal cancer patients not candidates for platinum retreatment (NItCHE-MITO33)	III	Recurrent	427	Arm A: Dostarlimab, NiraparibArm B: Chemotherapy (pegylated liposomal doxorubicin, paclitaxel, gemcitabine, topotecan, bevacizumab)	OS*PFSORRAEPRO	January 2025
NCT03598270	Platinum-based chemotherapy with Atezolizumab and Niraparib in patients with recurrent ovarian cancer	III	Recurrent	414	Niraparib + chemotherapy (gemcitabine, carboplatin, paclitaxel, pegylated liposomal doxorubicin) + /- atezolizumab	OS*PFSPFS2AEPROsORRDOR	January 2025
NCT04742075	Olaparib, Durvalumab and UV1 in relapsed ovarian cancer (DOVACC)	II	Recurrent	184	Arm A: OlaparibArm B: olaparib, durvalumabArm C: Olaparib, durvalumab, UV1	ORR*PROORR Safety	June 2026
NCT05231122	Pembrolizumab combined with bevacizumab with or without agonist Anti-CD40 CDX-1140 for the treatment of recurrent ovarian cancer	II	Recurrent	80	Pembrolizumab, bevacizumab, CDX-1140 (anti-CD40 agonist monoclonal antibody)	AE*ORR*PFSOSDCRQoL	January 2026
NCT04781088	Lenvatinib, Pembrolizumab, and Paclitaxel for treatment of recurrent endometrial, epithelial ovarian, fallopian and peritoneal cancer	II	Recurrent	38	Pembrolizumab, Lenvatinib, paclitaxel	ORR*AEPFS	February 2025
NCT03206047	Atezolizumab, Guadecitabine, and CDX-1401 Vaccine in treating patients with Recurrent Ovarian, Fallopian tube and peritoneal Cancer	II	Recurrent	75	Atezolizumab + Guadecitabine + CDX-1401 vaccine	AE*PFS*OSORRCA125 reductionDOR	March 2023
NCT02873962	A phase II study of Nivolumab/ Bevacizumab/Rucaparib	II	Recurrent	76	Nivolumab, bevacizumab, rucaparib	ORR*AE*PFSOSDOR	June 2024
NCT05092360	Phase III study of Nemvaleukin alfa in combination with pembrolizumab in patients with platinum-resistant epithelial ovarian cancer (ARTISTRY-7)	III	Recurrent	376	Arm A: Nemvaleukin-alfa plus pembrolizumabArm B: Nemavaleukin-alfaArm C: PembrolizumabArm D: Physician’s choice chemotherapy	PFS*ORROSDCRDORTTRCA125 AE	December 2026

ORR: Objective response rate; CRS: chemotherapy response score; PFS: progression-free survival; OS: overall survival; PRO: patient-reported outcomes; TTD: time to deterioration; pCR: pathological complete response; DOR: Duration of Response; AE: adverse events; CA125: Cancer Antigen 125; QoL: quality of life; PFS2: progression-free survival to second therapy; PD-L1: programmed cell death ligand 1; CPS: combined positive score; TTR: time to response. *primary endpoint.

The AMBITION study was the first biomarker-driven, targeted trial in heavily pre-treated platinum-resistant EOC, which included 70 patients^[97]^. The patients were allocated to receive combination therapy based on HRD and PD-L1 status determined on archival tumour sample. The patients were randomised to either olaparib and cediranib arm or olaparib or durvalumab arm. For HRD-positive patients or for HRD-negative patients, they were allocated to either durvalumab and ChT (either pegylated liposomal doxorubicin or topotecan or weekly paclitaxel) or durvalumab and tremelimumab and ChT. PD-L1 positivity was defined as 25% or more of expression via Ventana SP263 assay. The overall ORR was 37.1%, with the highest ORR of 50% observed in Olaparib-cediranib cohort, closely followed by olaparib-durvalumab cohort with an ORR of 42.9%, as excepted for HRD-negative cohorts, which had a low ORR ranging between 20%-33%. All treatment groups were manageable, with no treatment-related adverse events leading to discontinuation.

Currently, there are many ongoing trials of ICI with different combinations in EOC. These are listed in Table 1, although the list of trials included is not exhaustive.

## NOVEL TARGETS AND IMMUNE ENHANCEMENT IN OVARIAN CANCER

Unfortunately, it remains unclear as to whether ICI therapies will prove successful in improving clinical outcomes in EOC, and investigation of several novel therapeutic targets is currently underway.

### Antibody-drug conjugates

Structurally, antibody-drug conjugates (ADC) are comprised of a targeted monoclonal antibody attached to a cytotoxic payload through a linker molecule, where binding of the antibody to its target results in endocytosis and subsequent cytotoxic action in cancer cells^[98]^. Several molecules have shown promise in the treatment of EOC, as discussed below, although they remain highly variable in efficacy and rates of toxicity. Trials remain in progress for certain therapeutic targets in ovarian cancer, including tissue factor (tisotumab vedotin, NCT03657043) and mesothelin (anetumab ravtansine, NCT03587311).

### Mirvetuximab soravtansine

Folate receptor-alpha (FRα) is a cell surface protein frequently overexpressed in EOC in up to 81.5% of ovarian cancer tumours^[99]^. Mirvetuximab soravtansine targets this receptor and is linked to a maytansinoid DM4, which is a tubulin targeting agent. In November 2022, the US Food and Drug Administration approved Mirvetuximab soravtansine, a novel antibody-drug conjugate, for platinum-resistant EOC with FRα-positive disease based on phase III SORAYA study^[100]^. This study included 366 patients expressing FRα who received either mirvetuximab soravtansine or physician’s choice chemotherapy^[100]^. There was no PFS difference seen in either the overall population or FRα-high cohorts; however, the secondary outcomes (ORR, CA125 response, patient-reported outcomes) demonstrated significant improvement in Frα-high subgroups. The ORR was 24% compared to 10%, CA125 response was 53% compared to 25% and patient-reported outcome was superior with mirvetuximab soravtansine with 27% compared to 13%. There was no statistically significant difference in median OS in the overall population or in FRα-high subgroups. It also demonstrated a more manageable safety profile than chemotherapy, mainly resulting in low-grade, reversible ocular and gastrointestinal issues manageable with supportive interventions. Overall, there were fewer treatment-related grade 3 or higher adverse events (25.1% *vs.* 44%) and fewer treatment discontinuations (4.5% *vs.* 8.3%).

FRα as a therapeutic target has shown promise with reasonable adverse event rates, and it may be worthwhile looking for ways to increase the sensitivity of EOC cells to these molecules in future studies.

### NaPi2b targeting ADCs

NaPi2b is a cell surface sodium-dependent phosphate transporter and is overexpressed in select cancers including ovarian cancer. Two ADC molecules, lifastuzumab vedotin and upifitamab rilsodotin, have demonstrated modest efficacy in early-phase trials^[101,102]^ and several other studies are underway (NCT03319628, NCT04907968, NCT05329545).

### Epacadostat

Another investigational agent, Epacadostat, is a potential therapeutic in a number of tumour types, including ovarian cancer. Epacadostat is an Indoleamine 2,3-dioxygenase-1 (IDO1) inhibitor. IDO1 is a key regulator of immune tolerance in ovarian cancer, facilitating the breakdown of tryptophan and its mechanism, resulting in increased Tregs, myeloid-derived suppressor cells (MDSCs) with decreased tumour infiltration lymphocytes, and NK cells with upregulation of PD-1 in cytotoxic T cells overall leading to an immunosuppressive effect on TME. Overexpression of IDO1 has been associated with worse prognosis in various cancers,c and up to 56.7% of ovarian cancer has been identified to exhibit high IDO1 expression^[103]^. With evidence of preclinical studies demonstrating synergistic effects of epacadostat and immune checkpoint blockade, many studies have been conducted in combination with ICI. So far, results have been largely disappointing, with only moderate efficacy seen in the melanoma and renal cell carcinoma cohort in phase III study^[104]^. A phase II basket study examined the safety of epacadostat with pembrolizumab (NCT02178722) in 44 participants, including 37 with recurrent EOC. Unfortunately, a modest ORR of 8.1% was seen in this cohort. A further basket trial of epacadostat with nivolumab in solid tumours demonstrated a modest ORR of 14% and a disease control rate of 31% in a pre-treated ovarian cancer cohort of 29 patients^[105]^. Currently, there is a phase I trial (NCT02042430) examining the safety and tolerability of epacadostat prior to cytoreductive surgery for newly diagnosed EOC.

### Adavosertib

Adavosertib (AZD1775) is a Wee1 nuclear kinase inhibitor that has emerged as a potential compound able to regulate G2-M transition in the cell cycle and sensitise *TP53*-mutant cells to chemotherapy. Recent phase II trials in platinum-resistant or platinum-refractory settings demonstrated that when adavosertib was combined with gemcitabine, PFS was longer in the combination arm (median PFS 4.6 *vs.* 3 months)^[106]^. Another single-arm phase II study examining adavosertib with carboplatin demonstrated an ORR of 38% with a median PFS of 5.6 months^[107]^. Neither study demonstrated safety or toxicity issues, and side effects were reported to be manageable. Adavosertib is currently being examined with olaparib (NCT03579316) in recurrent EOC.

### Gemogenovatucel-T

Gemogenovatucel-T (Vigil) is a novel autologous tumour cell immunotherapy with multiple functions, including granulocyte-macrophage colony-stimulating factor (*GMCSF)* gene activation and TGF-ß1 and TGF-ß2 suppression through bi-functional short-hairpin RNA construct targeted to furin leading to generate a systemic immune response. Gemogenovatucel-T has been shown efficacy in phase IIb trial with a recurrence-free survival of 11.5 months (95% CI: 7.5-NR) compared to 8.4 months (95% CI: 7.9-15.5) in placebo arm with significant benefit in *BRCA-*wt patients with HR 0.69 (90% CI 0.44-1.07, one-sided *P* = 0.078)^[108]^_._ Further post-hoc subgroup analysis demonstrated recurrent-free survival and OS benefit in *BRCA-*wt, homologous recombination proficient (HRP) patient population, an effect demonstrated out to up to 3 years^[109,110]^_._ It remains unclear as to why this drug was particularly efficacious in the *BRCA-*wt population, but ongoing biomarker studies in EOC patients may reveal further answers in the future.

Gemogenovatucel-T and durvalumab combination were investigated in 5 *BRCA-*wild-type recurrent/refractory EOC in a pilot basket study alongside triple-negative breast cancer patients^[111]^. Median PFS was 7.1 months with median OS not reached with greater benefit in PD-L1 expressing tumours *(n* = 8, HR 0.304, 95%CI: 0.0593-1.56, 1-sided *P* = 0.4715). There were three grade 3 treatment-related adverse events, all related to durvalumab, therefore demonstrating this combination is well-tolerated and has promising clinical activity in recurrent/refractory EOC cohort warranting further investigation_._

### Nemvaleukin-alfa

Nemvaleukin-alfa is an engineered cytokine binding selectively to intermediate affinity IL-2 receptors (IL-2R), leading to downstream activation of CD8-positive T-cells and NK cells and limited activation of regulatory T cells. Its preliminary activity was demonstrated through the ARTISTRY-1 study (NCT02799095), a phase I/II basket study that included an ovarian cancer cohort of 14 patients in combination with pembrolizumab (ORR 29%) with side effects most commonly being cytopenias^[112]^. A four-arm, randomised, phase III study is currently underway (NCT05092360) with the same combination using physician’s choice chemotherapy as the main comparator arm.

### Nanoparticle-based combination immunotherapy

An active field of therapeutic development currently is in the development of nanotechnology with the development of nanoparticles (NP)^[113]^_._ These act as drug delivery vehicles capable of providing localised and targeted therapeutic effects of immunotherapy with minimal toxicity when administered due to their targeted nature^[113]^. The technology is currently in its early days and multiple trials are being undertaken to evaluate its therapeutic value, particularly in relation to NP-bound chemotherapy^[113]^.

### Cancer vaccines

As referenced in Table 1 (NCT0320047), cancer vaccines are currently being investigated in ovarian cancer to ‘sensitise’ the immune system to cancer antigens with the aim of inducing an immune response. Although there have been a plethora of studies investigating various formulations^[114]^, the clinical evidence remains very much in its infancy in treating ovarian cancer, although one recent phase II study in 10 heavily pre-treated, platinum-resistant EOC combining maveropepimut-S, a DPX-platform-delivered peptide cancer vaccine of the antigen survivin, with pembrolizumab and oral cyclophosphamide met its primary efficacy endpoint with 10% partial response and 20% stable disease for more than 12 weeks^[115]^.

## CONCLUSION

Despite many advancements in understanding tumour biology and drug development within the last few decades, EOC remains a challenging disease to treat, with unacceptably high recurrence and mortality rates. Improved understanding of tumour biology and resistance mechanisms to current therapy, as well as the intricate interplay between tumour, TME and immune system, has paved the way for the development of immunotherapy. However, despite initial aspirations of transforming EOC from “cold” into “hot”, immunogenic tumours with utilisation of ICI, this has been largely disappointing with modest clinical activity, suggesting that there are inherent resistance mechanisms at play within the EOC microenvironment. This review has attempted to highlight key players involved in the immune system and TME in the context of EOC; however, it is by no means a complete or exhaustive description of the myriad of key components and processes which make up the complex ecosystem.

Combining immunotherapies with other drug classes has shown early promise, but larger randomised trials are required to clearly define the role of ICI therapy in the EOC treatment landscape, if any. Importantly further studies in the mechanistic activity of immunomodulators and their interaction with ICI, TME and immune system to understand the enhanced efficacy of ICI combination therapy will be an important area of research in the future. Novel therapeutic classes such as antibody-drug conjugates and tumour-directed vaccines are of growing interest, and future studies involving biomarker strategies and robust research methods will be needed to determine which EOC patients will ultimately gain the most benefit from specific therapeutic combinations, with the ultimate goal of improving patient survival and quality of life.

## DECLARATIONS

### Copyright

© The Author(s) 2023.

## References

[1] Sung H, Ferlay J, Siegel RL (2021). Global cancer statistics 2020: GLOBOCAN estimates of incidence and mortality worldwide for 36 cancers in 185 countries. CA Cancer J Clin.

[2] https://publications.iarc.fr/Book-And-Report-Series/Who-Classification-Of-Tumours/Female-Genital-Tumours-2020.

[3] (2011). Cancer Genome Atlas Research Network. Integrated genomic analyses of ovarian carcinoma. Nature.

[4] Peres LC, Cushing-Haugen KL, Köbel M (2019). Invasive epithelial ovarian cancer survival by histotype and disease stage. J Natl Cancer Inst.

[5] Zhang S, Royer R, Li S (2011). Frequencies of BRCA1 and BRCA2 mutations among 1,342 unselected patients with invasive ovarian cancer. Gynecol Oncol.

[6] Norquist BM, Harrell MI, Brady MF (2016). Inherited mutations in women with ovarian carcinoma. JAMA Oncol.

[7] Pal T, Permuth-Wey J, Betts JA (2005). BRCA1 and BRCA2 mutations account for a large proportion of ovarian carcinoma cases. Cancer.

[8] Poveda A, Floquet A, Ledermann JA, SOLO2/ENGOT-Ov21 investigators (2021). Olaparib tablets as maintenance therapy in patients with platinum-sensitive relapsed ovarian cancer and a BRCA1/2 mutation (SOLO2/ENGOT-Ov21): a final analysis of a double-blind, randomised, placebo-controlled, phase 3 trial. Lancet Oncol.

[9] Banerjee S, Moore KN, Colombo N (2021). Maintenance olaparib for patients with newly diagnosed advanced ovarian cancer and a BRCA mutation (SOLO1/GOG 3004): 5-year follow-up of a randomised, double-blind, placebo-controlled, phase 3 trial. Lancet Oncol.

[10] Ledermann JA, Raja FA, Fotopoulou C, Gonzalez-Martin A, Colombo N, Sessa C, ESMO Guidelines Working Group (2013). Newly diagnosed and relapsed epithelial ovarian carcinoma: ESMO clinical practice guidelines for diagnosis, treatment and follow-up. Ann Oncol.

[11] Cress RD, Chen YS, Morris CR, Petersen M, Leiserowitz GS (2015). Characteristics of long-term survivors of epithelial ovarian cancer. Obstet Gynecol.

[12] Bookman MA, Brady MF, McGuire WP (2009). Evaluation of new platinum-based treatment regimens in advanced-stage ovarian cancer: a Phase III Trial of the Gynecologic Cancer Intergroup. J Clin Oncol.

[13] Böhm S, Faruqi A, Said I (2015). Chemotherapy Response Score: Development and validation of a system to quantify histopathologic response to neoadjuvant chemotherapy in tubo-ovarian high-grade serous carcinoma. J Clin Oncol.

[14] Cohen PA, Powell A, Böhm S, HGSC CRS Collaborative Network (Supplementary 1) (2020). Corrigendum to “Pathological chemotherapy response score is prognostic in tubo-ovarian high-grade serous carcinoma: a systematic review and meta-analysis of individual patient data”[Gynecol. Oncol. 154 (2019) 441-448].. Gynecol Oncol.

[15] Burger RA, Brady MF, Bookman MA, Gynecologic Oncology Group (2011). Incorporation of bevacizumab in the primary treatment of ovarian cancer. N Engl J Med.

[16] Tewari KS, Burger RA, Enserro D (2019). Final overall survival of a randomized trial of bevacizumab for primary treatment of ovarian cancer. J Clin Oncol.

[17] Perren TJ, Swart AM, Pfisterer J, ICON7 Investigators (2011). A phase 3 trial of bevacizumab in ovarian cancer. N Engl J Med.

[18] Friedlander M, Gebski V, Gibbs E (2018). Health-related quality of life and patient-centred outcomes with olaparib maintenance after chemotherapy in patients with platinum-sensitive, relapsed ovarian cancer and a BRCA1/2 mutation (SOLO2/ENGOT Ov-21): a placebo-controlled, phase 3 randomised trial. Lancet Oncol.

[19] Moore K, Colombo N, Scambia G (2018). Maintenance olaparib in patients with newly diagnosed advanced ovarian cancer. N Engl J Med.

[20] Sugiyama T, Kamura T, Kigawa J (2000). Clinical characteristics of clear cell carcinoma of the ovary: a distinct histologic type with poor prognosis and resistance to platinum-based chemotherapy. Cancer.

[21] Rottenberg S, Disler C, Perego P (2021). The rediscovery of platinum-based cancer therapy. Nat Rev Cancer.

[22] Domcke S, Sinha R, Levine DA, Sander C, Schultz N (2013). Evaluating cell lines as tumour models by comparison of genomic profiles. Nat Commun.

[23] Patch AM, Christie EL, Etemadmoghadam D, Australian Ovarian Cancer Study Group (2015). Whole-genome characterization of chemoresistant ovarian cancer. Nature.

[24] Burdett NL, Willis MO, Alsop K (2023). Multiomic analysis of homologous recombination-deficient end-stage high-grade serous ovarian cancer. Nat Genet.

[25] Menghi F, Banda K, Kumar P (2022). Genomic and epigenomic BRCA alterations predict adaptive resistance and response to platinum-based therapy in patients with triple-negative breast and ovarian carcinomas. Sci Transl Med.

[26] Lheureux S, Gourley C, Vergote I, Oza AM (2019). Epithelial ovarian cancer. Lancet.

[27] Larkin J, Chiarion-Sileni V, Gonzalez R (2019). Five-Year survival with combined Nivolumab and Ipilimumab in advanced melanoma. N Engl J Med.

[28] Reck M, Rodríguez-Abreu D, Robinson AG, KEYNOTE-024 Investigators (2016). Pembrolizumab versus chemotherapy for PD-L1-positive non-small-cell lung cancer. N Engl J Med.

[29] André T, Shiu KK, Kim TW, KEYNOTE-177 Investigators (2020). Pembrolizumab in microsatellite-instability-high advanced colorectal cancer. N Engl J Med.

[30] Giuntoli RL 2nd, Webb TJ, Zoso A (2009). Ovarian cancer-associated ascites demonstrates altered immune environment: implications for antitumor immunity. Anticancer Res.

[31] Toker A, Nguyen LT, Stone SC (2018). Regulatory T cells in ovarian cancer are characterized by a highly activated phenotype distinct from that in melanoma. Clin Cancer Res.

[32] Larionova I, Tuguzbaeva G, Ponomaryova A (2020). Tumor-associated macrophages in human breast, colorectal, lung, ovarian and prostate cancers. Front Oncol.

[33] Santoiemma PP, Reyes C, Wang LP (2016). Systematic evaluation of multiple immune markers reveals prognostic factors in ovarian cancer. Gynecol Oncol.

[34] Garsed DW, Pandey A, Fereday S (2022). The genomic and immune landscape of long-term survivors of high-grade serous ovarian cancer. Nat Genet.

[35] Brahmer JR, Tykodi SS, Chow LQM (2012). Safety and activity of anti-PD-L1 antibody in patients with advanced cancer. N Engl J Med.

[36] Disis ML, Taylor MH, Kelly K (2019). Efficacy and safety of avelumab for patients with recurrent or refractory ovarian cancer: Phase 1b Results From the JAVELIN Solid Tumor Trial. JAMA Oncol.

[37] Varga A, Piha-Paul S, Ott PA (2019). Pembrolizumab in patients with programmed death ligand 1-positive advanced ovarian cancer: analysis of KEYNOTE-028. Gynecol Oncol.

[38] Monk BJ, Colombo N, Oza AM (2021). Chemotherapy with or without avelumab followed by avelumab maintenance versus chemotherapy alone in patients with previously untreated epithelial ovarian cancer (JAVELIN Ovarian 100): an open-label, randomised, phase 3 trial. Lancet Oncol.

[39] Moore KN, Bookman M, Sehouli J (2021). Atezolizumab, Bevacizumab, and Chemotherapy for newly diagnosed stage III or IV ovarian cancer: placebo-controlled randomized phase III trial (IMagyn050/GOG 3015/ENGOT-OV39). J Clin Oncol.

[40] Kurman RJ, Shih IeM (2016). The dualistic model of ovarian carcinogenesis: revisited, revised, and expanded. Am J Pathol.

[41] Goode EL, Block MS, Kalli KR, Ovarian Tumor Tissue Analysis (OTTA) Consortium (2017). Dose-response association of CD8+ tumor-infiltrating lymphocytes and survival time in high grade serous ovarian cancer. JAMAOncol.

[42] Meagher NS, Hamilton P, Milne K (2023). Profiling the immune landscape in mucinous ovarian carcinoma. Gynecol Oncol.

[43] Boussios S, Rassy E, Moschetta M (2022). BRCA mutations in ovarian and prostate cancer: bench to bedside. Cancers.

[44] Vázquez-García I, Uhlitz F, Ceglia N (2022). Ovarian cancer mutational processes drive site-specific immune evasion. Nature.

[45] Hwang WT, Adams SF, Tahirovic E, Hagemann IS, Coukos G (2012). Prognostic significance of tumor-infiltrating T cells in ovarian cancer: a meta-analysis. Gynecol Oncol.

[46] Maimela NR, Liu S, Zhang Y (2019). Fates of CD8+ T cells in tumor microenvironment. Comput Struct Biotechnol J.

[47] Labani-Motlagh A, Ashja-Mahdavi M, Loskog A (2020). The tumor microenvironment: a milieu hindering and obstructing antitumor immune responses. Front Immunol.

[48] Kandalaft LE, Dangaj Laniti D, Coukos G (2022). Immunobiology of high-grade serous ovarian cancer: lessons for clinical translation. Nat Rev Cancer.

[49] Santoiemma PP, Powell DJ Jr (2015). Tumor infiltrating lymphocytes in ovarian cancer. Cancer Biol Ther.

[50] Curiel TJ, Coukos G, Zou L (2004). Specific recruitment of regulatory T cells in ovarian carcinoma fosters immune privilege and predicts reduced survival. Nat Med.

[51] Barnett JC, Bean SM, Whitaker RS (2010). Ovarian cancer tumor infiltrating T-regulatory (T(reg)) cells are associated with a metastatic phenotype. Gynecol Oncol.

[52] Pölcher M, Braun M, Friedrichs N (2010). Foxp3(+) cell infiltration and granzyme B(+)/Foxp3(+) cell ratio are associated with outcome in neoadjuvant chemotherapy-treated ovarian carcinoma. Cancer Immunol Immunother.

[53] Groth C, Hu X, Weber R (2019). Immunosuppression mediated by myeloid-derived suppressor cells (MDSCs) during tumour progression. Br J Cancer.

[54] Nagaraj S, Gabrilovich DI (2012). Regulation of suppressive function of myeloid-derived suppressor cells by CD4+ T cells. Semin Cancer Biol.

[55] Zhang L, Conejo-Garcia JR, Katsaros D (2003). Intratumoral T cells, recurrence, and survival in epithelial ovarian cancer. N Engl J Med.

[56] Hamanishi J, Mandai M, Iwasaki M (2007). Programmed cell death 1 ligand 1 and tumor-infiltrating CD8+ T lymphocytes are prognostic factors of human ovarian cancer. Proc Natl Acad Sci U S A.

[57] Matsuzaki J, Gnjatic S, Mhawech-Fauceglia P (2010). Tumor-infiltrating NY-ESO-1-specific CD8+ T cells are negatively regulated by LAG-3 and PD-1 in human ovarian cancer. Proc Natl Acad Sci U S A.

[58] Francisco LM, Sage PT, Sharpe AH (2010). The PD-1 pathway in tolerance and autoimmunity. Immunol Rev.

[59] Chen R, Zinzani PL, Fanale MA, KEYNOTE-087 (2017). Phase II study of the efficacy and safety of pembrolizumab for relapsed/refractory classic hodgkin lymphoma. J Clin Oncol.

[60] Marabelle A, Le DT, Ascierto PA (2020). Efficacy of pembrolizumab in patients with noncolorectal high microsatellite instability/mismatch repair-deficient cancer: results from the phase II KEYNOTE-158 study. J Clin Oncol.

[61] Le DT, Kim TW, Van Cutsem E (2020). Phase II open-label study of pembrolizumab in treatment-refractory, microsatellite instability-high/mismatch repair-deficient metastatic colorectal cancer: KEYNOTE-164. J Clin Oncol.

[62] Liu YT, Sun ZJ (2021). Turning cold tumors into hot tumors by improving T-cell infiltration. Theranostics.

[63] Matulonis UA, Shapira-Frommer R, Santin AD (2019). Antitumor activity and safety of pembrolizumab in patients with advanced recurrent ovarian cancer: results from the phase II KEYNOTE-100 study. Ann Oncol.

[64] Pujade-Lauraine E, Fujiwara K, Ledermann JA (2021). Avelumab alone or in combination with chemotherapy versus chemotherapy alone in platinum-resistant or platinum-refractory ovarian cancer (JAVELIN Ovarian 200): an open-label, three-arm, randomised, phase 3 study. Lancet Oncol.

[65] Hamanishi J, Takeshima N, Katsumata N (2021). Nivolumab versus gemcitabine or pegylated liposomal doxorubicin for patients with platinum-resistant ovarian cancer: open-label, randomized trial in japan (NINJA). J Clin Oncol.

[66] Hamanishi J, Mandai M, Ikeda T (2015). Safety and antitumor activity of anti-PD-1 antibody, nivolumab, in patients with platinum-resistant ovarian cancer. J Clin Oncol.

[67] Tan DSP, Choi CH, Ngoi N (2022). A multicenter phase II randomized trial of durvalumab (D) versus physician’s choice chemotherapy (PCC) in patients (pts) with recurrent ovarian clear cell adenocarcinoma (MOCCA/APGOT-OV2/GCGS-OV3). J Clin Oncol.

[68] Zamarin D, Burger RA, Sill MW (2020). Randomized phase II trial of nivolumab versus nivolumab and ipilimumab for recurrent or persistent ovarian cancer: an NRG oncology study. J Clin Oncol.

[69] Yamamoto S, Konishi I, Mandai M (1997). Expression of vascular endothelial growth factor (VEGF) in epithelial ovarian neoplasms: correlation with clinicopathology and patient survival, and analysis of serum VEGF levels. Br J Cancer.

[70] Pujade-Lauraine E, Hilpert F, Weber B (2014). Bevacizumab combined with chemotherapy for platinum-resistant recurrent ovarian cancer: The AURELIA open-label randomized phase III trial. J Clin Oncol.

[71] Voron T, Colussi O, Marcheteau E (2015). VEGF-A modulates expression of inhibitory checkpoints on CD8+ T cells in tumors. J Exp Med.

[72] Voron T, Marcheteau E, Pernot S (2014). Control of the immune response by pro-angiogenic factors. Front Oncol.

[73] Facciabene A, Peng X, Hagemann IS (2011). Tumour hypoxia promotes tolerance and angiogenesis via CCL28 and T(reg) cells. Nature.

[74] Li B, Lalani AS, Harding TC (2006). Vascular endothelial growth factor blockade reduces intratumoral regulatory T cells and enhances the efficacy of a GM-CSF-secreting cancer immunotherapy. Clin Cancer Res.

[75] Moroney JW, Powderly J, Lieu CH (2020). Safety and clinical activity of atezolizumab plus bevacizumab in patients with ovarian cancer: a phase ib study. Clin Cancer Res.

[76] Liu JF, Herold C, Gray KP (2019). Assessment of combined nivolumab and bevacizumab in relapsed ovarian cancer: a phase 2 clinical trial. JAMA Oncol.

[77] Gonzalez-Martin A, Chung HC, Saada-Bouzid E (2020). 297 Leap-005: evaluating the safety and efficacy of Lenvatinib and pembrolizumab in patients previously treated for ovarian cancer, a multi-cohort phase 2 study. Int J Gynecol Cancer.

[78] Coward J, Gao B, de Silva IP https://www.beigenemedical.com/CongressDocuments/Goh_BGB-900-103_AACR_Presentation_2021.pdf.

[79] Coward J, Frentzas S, Mislang A (2021). 427 Efficacy and safety of AK112, an anti-PD-1/VEGF-A bispecific antibody, in patients with platinum-resistant/refractory epithelial ovarian cancer in a Phase 1 study. J Immunother Cancer.

[80] McMullen M, Karakasis K, Madariaga A, Oza AM (2020). Overcoming platinum and PARP-Inhibitor resistance in ovarian cancer. Cancers.

[81] Vaidyanathan A, Sawers L, Gannon AL (2016). ABCB1 (MDR1) induction defines a common resistance mechanism in paclitaxel- and olaparib-resistant ovarian cancer cells. Br J Cancer.

[82] Pettitt SJ, Krastev DB, Brandsma I (2018). Genome-wide and high-density CRISPR-Cas9 screens identify point mutations in PARP1 causing PARP inhibitor resistance. Nat Commun.

[83] Weigelt B, Comino-Méndez I, de Bruijn I (2017). Diverse BRCA1 and BRCA2 reversion Mutations in circulating cell-free DNA of therapy-resistant breast or ovarian cancer. Clin Cancer Res.

[84] Lin KK, Harrell MI, Oza AM (2019). BRCA reversion mutations in circulating tumor DNA predict primary and acquired resistance to the PARP inhibitor rucaparib in high-grade ovarian carcinoma. Cancer Discov.

[85] Kondrashova O, Nguyen M, Shield-Artin K, AOCS study group (2017). Secondary somatic mutations restoring RAD51C and RAD51D associated with acquired resistance to the PARP Inhibitor rucaparib in high-grade ovarian carcinoma. Cancer Discov.

[86] Kondrashova O, Topp M, Nesic K, Australian Ovarian Cancer Study (AOCS) (2018). Methylation of all BRCA1 copies predicts response to the PARP inhibitor rucaparib in ovarian carcinoma. Nat Commun.

[87] Lheureux S, Bruce JP, Burnier JV (2017). Somatic BRCA1/2 Recovery as a resistance mechanism after exceptional response to poly (ADP-ribose) polymerase inhibition. J Clin Oncol.

[88] Lee EK, Konstantinopoulos PA (2020). PARP inhibition and immune modulation: scientific rationale and perspectives for the treatment of gynecologic cancers. Ther Adv Med Oncol.

[89] Zhang Y, Cui Q, Xu M, Liu D, Yao S, Chen M (2022). Current advances in PD-1/PD-L1 blockade in recurrent epithelial ovarian cancer. Front Immunol.

[90] Jiao S, Xia W, Yamaguchi H (2017). PARP Inhibitor Upregulates PD-L1 expression and enhances cancer-associated immunosuppression. Clin Cancer Res.

[91] Wang Y, Zheng K, Xiong H (2021). PARP Inhibitor upregulates PD-L1 expression and provides a new combination therapy in pancreatic cancer. Front Immunol.

[92] Konstantinopoulos PA, Waggoner S, Vidal GA (2019). Single-arm phases 1 and 2 trial of niraparib in combination with pembrolizumab in patients with recurrent platinum-resistant ovarian carcinoma. JAMA Oncol.

[93] Drew Y, de Jonge M, Hong S (2018). An open-label, phase II basket study of olaparib and durvalumab (MEDIOLA): results in germline BRCA -mutated ( gBRCA m) platinum-sensitive relapsed (PSR) ovarian cancer (OC). Gynecol Oncol.

[94] Zimmer AS, Nichols E, Cimino-Mathews A (2019). A phase I study of the PD-L1 inhibitor, durvalumab, in combination with a PARP inhibitor, olaparib, and a VEGFR1-3 inhibitor, cediranib, in recurrent women's cancers with biomarker analyses. J Immunother Cancer.

[95] Freyer G, Floquet A, Tredan O (2021). 733P Bevacizumab (Bev), olaparib (Ola) and durvalumab (Durva) in patients with recurrent advanced ovarian cancer (AOC): The GINECO BOLD study. Ann Oncol.

[96] Liu J, Gaillard S, Hendrickson AW (2021). An open-label phase II study of dostarlimab (TSR-042), bevacizumab (bev), and niraparib combination in patients (pts) with platinum-resistant ovarian cancer (PROC): cohort A of the OPAL trial. Gynecol Oncol.

[97] Lee JY, Kim BG, Kim JW, Korean Gynecologic Oncology Group (KGOG) investigators (2022). Biomarker-guided targeted therapy in platinum-resistant ovarian cancer (AMBITION; KGOG 3045): a multicentre, open-label, five-arm, uncontrolled, umbrella trial. J Gynecol Oncol.

[98] Manzano A, Ocaña A (2020). Antibody-drug conjugates: a promising novel therapy for the treatment of ovarian cancer. Cancers.

[99] Kalli KR, Oberg AL, Keeney GL (2008). Folate receptor alpha as a tumor target in epithelial ovarian cancer. Gynecol Oncol.

[100] Moore KN, Oza AM, Colombo N (2021). Phase III, randomized trial of mirvetuximab soravtansine versus chemotherapy in patients with platinum-resistant ovarian cancer: primary analysis of FORWARD I. Ann Oncol.

[101] Banerjee S, Oza AM, Birrer MJ (2018). Anti-NaPi2b antibody-drug conjugate lifastuzumab vedotin (DNIB0600A) compared with pegylated liposomal doxorubicin in patients with platinum-resistant ovarian cancer in a randomized, open-label, phase II study. Ann Oncol.

[102] Hays J, Werner T, Lakhani N (2022). TP024/#446 Upgrade: phase 1 combination trial of the NAPI2B-directed antibody drug conjugate (ADC) upifitamab rilsodotin (UPRI; XMT-1536) in patients with ovarian cancer. Int J Gynecol Cancer.

[103] Inaba T, Ino K, Kajiyama H (2009). Role of the immunosuppressive enzyme indoleamine 2,3-dioxygenase in the progression of ovarian carcinoma. Gynecol Oncol.

[104] Mitchell TC, Hamid O, Smith DC (2018). Epacadostat plus pembrolizumab in patients with advanced solid tumors: phase i results from a multicenter, open-label phase i/ii trial (ECHO-202/KEYNOTE-037). J Clin Oncol.

[105] Perez RP, Riese MJ, Lewis KD (2017). Epacadostat plus nivolumab in patients with advanced solid tumors: preliminary phase I/II results of ECHO-204. J Clin Oncol.

[106] Lheureux S, Cristea MC, Bruce JP (2021). Adavosertib plus gemcitabine for platinum-resistant or platinum-refractory recurrent ovarian cancer: a double-blind, randomised, placebo-controlled, phase 2 trial. Lancet.

[107] Embaby A, Geenen JJ, Kutzera J (2022). Adavosertib in combination with carboplatin in advanced TP53- mutated platinum-resistant ovarian cancer. J Clin Oncol.

[108] Rocconi RP, Grosen EA, Ghamande SA (2020). Gemogenovatucel-T (Vigil) immunotherapy as maintenance in frontline stage III/IV ovarian cancer (VITAL): a randomised, double-blind, placebo-controlled, phase 2b trial. Lancet Oncol.

[109] Rocconi RP, Ghamande SA, Barve MA, The vigil team (2021). Maintenance vigil immunotherapy in newly diagnosed advanced ovarian cancer: efficacy assessment of homologous recombination proficient (HRP) patients in the phase IIb VITAL trial. J Clin Oncol.

[110] Walter A, Rocconi RP, Monk BJ (2021). Gemogenovatucel-T (Vigil) maintenance immunotherapy: 3-year survival benefit in homologous recombination proficient (HRP) ovarian cancer. Gynecol Oncol.

[111] Barve M, Aaron P, Manning L (2022). Pilot study of combination gemogenovatucel-T (vigil) and durvalumab in women with relapsed BRCA-wt triple-negative breast or ovarian cancer. Clin Med Insights Oncol.

[112] Vaishampayan UN, Tomczak P, Muzaffar J (2022). Nemvaleukin alfa monotherapy and in combination with pembrolizumab in patients (pts) with advanced solid tumors: ARTISTRY-1. J Clin Oncol.

[113] Nadukkandy AS, Ganjoo E, Singh A, Dinesh Kumar L (2022). Tracing new landscapes in the arena of nanoparticle-based cancer immunotherapy. Front Nanotechnol.

[114] Chow S, Berek JS, Dorigo O (2020). Development of therapeutic vaccines for ovarian cancer. Vaccines.

[115] Veneziani A, Lheureux S, Alqaisi H (2022). Pembrolizumab, maveropepimut-S, and low-dose cyclophosphamide in advanced epithelial ovarian cancer: Results from phase 1 and expansion cohort of PESCO trial. J Clin Oncol.

